# Quality of life in patients with hereditary haemorrhagic telangiectasia (HHT)

**DOI:** 10.1186/s12955-017-0586-z

**Published:** 2017-01-23

**Authors:** Roberto Zarrabeitia, Concepción Fariñas-Álvarez, Miguel Santibáñez, Blanca Señaris, Ana Fontalba, Luisa María Botella, José Antonio Parra

**Affiliations:** 1 Department of Internal Medicine (Hospital Sierrallana) and Centro de Investigación Biomédica en red de Enfermedades Raras (CIBERER) and Instituto de Investigación Valdecilla (IDIVAL), Torrelavega, Cantabria Spain; 2 Quality Unit, Hospital Universitario Marqués de Valdecilla, Santander, Cantabria Spain; 3 University of Cantabria, Santander, Spain; 4 ENT Department, Hospital Sierrallana, Torrelavega, Cantabria Spain; 5 Department of Genetics, Hospital Universitario Marqués de Valdecilla, Santander, Cantabria Spain; 6 Centro de Investigaciones Biológicas, Consejo Superior de Investigaciones Científicas (CSIC) and Centro de Investigación Biomédica en red de Enfermedades Raras (CIBERER), Madrid, Spain; 7 Department of Radiology, Hospital Universitario Marqués de Valdecilla and Instituto de Investigación Valdecilla (IDIVAL), Santander, Spain

**Keywords:** HHT, Hereditary hemorrhagic telangiectasia, Rendu Osler, Euroqol, Qol

## Abstract

**Background:**

There are very few studies about general quality of life parameters, standards for the description of health status and comparison with general population data on patients with Hereditary hemorrhagic telangiectasia (HHT), a rare disease in which epistaxis is a cardinal symptom.

**Purpose:**

To assess the quality of life in a population of Spanish patients with HHT and compare it with the general population.

**Design and methods:**

Between January 1^st^ 2005 and December 31^st^ 2013, 187 adult patients diagnosed with HHT who were admitted to the HHT Unit of the Hospital Sierrallana, completed on their first visit, the EuroQol 5D-3L (five dimensions and three levels) quality of life descriptive test and the visual analog scale (VAS). The numerical social index value was also determined and the subjective effect of the nasal epistaxis on their quality of life was estimated classified as mild, moderate or severe.

**Results:**

Patients with HHT had greater problems than the general population in the five dimensions of the EuroQol 5D-3L, particularly considering pain/discomfort and anxiety/depression. In the VAS and the social index value, patients with HHT also scored lower than the general population, particularly older patients, males, and patients with HHT2. They also had values similar to those of populations with chronic illnesses. The subjective perception of the severity of epistaxis correlated strongly with the VAS and social index values.

**Conclusions:**

The quality of life of patients with HHT, estimated using the EuroQol 5D-3L scale, is affected across all dimensions. The scores are similar to those seen in cases of other chronic diseases. Older patients, males and the carriers of the *ACVRL1* mutation generally have worse scores on these scales. The VAS and the social index value are index that correlate well with the severity of the clinical symptoms associated mainly with epistaxis.

## Background

Hereditary hemorrhagic telangiectasia (HHT) or Osler-Weber-Rendu syndrome (ICD 9 448.0 [1] / ICD 10 178.0 [2] / ORPHA774) is an autosomal dominant genetic disease. It is characterized by the appearance of anomalous vascular structures: telangiectasias (small in size) or arteriovenous malformations (AVM). The latter are complex and larger in size. These vascular anomalies may appear in any organ and are responsible for the morbidity and mortality associated with this disease. The symptoms are variable in their penetrance, and are affected by a number of factors, including but not limited to age, sex and genotype. Epistaxis is the most frequent clinical manifestation: up to 96% of patients may exhibit nasal bleeding and it is generally the first symptom to appear [3]. HHT is classified as a rare or minority disease since its average prevalence is estimated at 1:5000–1:8000 [4], still remaining under-diagnosed [5], and associated with a significant morbidity and mortality [6]. At this moment there is no definitive cure for this disease.

Various genes involved in the development of the disease have been identified (Table 1).

**Table 1:** Genes involved in HHT

HHT	OMIM	Chromosome	Gene	Protein
HHT 1	#187300	9	*ENG*	endoglin
HHT 2	#600376	12	*ACVRL1*	ALK1
HTJP	#175050	18	*MADH4*	Smad 4
HHT 3?		5		
HHT 4?		7		
SMCMA		14	*BMP9 (GDF2)*	BMP9

The diagnosis of the illness is based on the Curaçao’s diagnostic criteria [7, 8]. These are: epistaxis, telangiectasias, dominant familial aggregation and effects on internal organs. At least three must observed in order to confirm a diagnosis. Otherwise, a diagnosis can be performed by identifying, via molecular study, a suspected causative mutation.

There are few studies that provide reference population data, standards for the description of health status, or general quality of life parameters in HHT patients. Furthermore, the majority of publications apply the SF-36 scale (original or abbreviated). At the present time, no quality of life scale has been validated specifically for HHT but a questionare of specific symptoms linked with HHT performed on a sample of German patients within a profile of quality of life of chronically ill patients survey [9]. The HHT Unit at the Hospital Sierrallana has been listed by the International HHT Foundation (www.hht.org) as international excellence center and it has functioned as a reference unit for this pathology in Spain since 2002. With authorization from theEuroQol Group (http://www.euroqol.org/), the Unit has applied the Euroquol-5D-3L scale since 2005 [10]. This scale is a generic, standardized tool that is being developed internationally since 1990. Euroquol-5D-3L is used to describe and assess the self-perceived quality of life in the area of health.

Therefore, the aim of our study was to investigate the quality of life in a population of Spanish patients with HHT and compare it with the general population.

## Population and methods

The study was performed at the Hospital Sierrallana (Torrelavega, Cantabria, Spain). The HHT Unit of this hospital serves as a reference unit for the population of Cantabria and Spain. Accreditation is pending, according to legislation governing the appointment of reference centers and units (Royal Decree 1302/2006). According to census figures published on the 1^st^ of January 2014 by the Spanish National Statistics Institute, INE), Cantabria had 587,686 inhabitants and the population of Spain was 46,507,760.

This was a descriptive, cross-sectional, observational study and included adult patients treated at the HHT Unit at the Hospital Sierrallana (General Medicine) diagnosed with HHT (clinical and/or genetic) between 2005 and 2013, who were asked to complete the EuroQol 5D-3L quality of life scale upon their first admission or consultation as well as provide a subjective valuation of the impact that epistaxis had on their health status (these last valuation was categorized as mild, moderate or severe based on self perceived impairment for normal life compared with an ideal status).

The EuroQol 5D consists of two parts. The first part is a descriptive system of health status. The system allocates a numerical 5-digit code. Each digit oscillates from 1 to 3 (no problems, some problems, and significant problems, respectively). The following dimensions were rated: mobility, self-care, usual activities, pain/discomfort and anxiety/depression. The three level version is used in the HHT Unit as this was the only version available when the system was first introduced. A five level version has also been available since 2009, however. From this descriptive system a social index value is obtained using a formula calculated according to the individual studies for the population of each country or region in which the best health status would be assigned a score of “1”, for full health. The worst health status would be death, which would be assigned a score of “0” [10].

The second part of the EuroQol 5D is a visual analog scale (VAS or EuroQol thermometer) that automatically assigns the patient a score between 0 and 100 (where 0 is the worst health status imaginable and 100 the best) in a vertical bar indicating a score for their perceived quality of life.

The following variables were analyzed:

- EuroQol code (descriptive, numerical qualitative variable)
- EuroQol social index value (continuous, quantitative variable)
- EuroQol VAS (continuous, quantitative variable)
- Subjective perception of general health status regarding impact of epistaxis (descriptive, categorical, qualitative variable) based on self perceived impairment for normal life compared with an ideal status).

The EuroQol 5D profile was dichotomized into ‘no problems’ and ‘moderate/extreme problems’.

To compare quality of life in HHT patients with general population we used data of the Spanish national health survey (ENSE) performed in 2011/2012 based on a sample of 26,502 people and data from the Navarre general population health survey of 1995 (*n* = 3000) [11]. Aditionally data from the Catalonian health survey (ESCA) of 1995 (*n* = 12,445 people) [12] was used both to compare HHT patients with general population and with patients with chronic diseases.

### Statistical analysis

Data were summarized as mean and standard deviation (SD) and median with interquartile range (IQR: p25-p75) for continuous variables, and frequency (%) for categorical variables. Statistical analysis was performed using two-tailed *χ*2 and Fisher’s exact test when necessary, one-way ANOVA test or Mann–Whitney *U* test or Kruskal-Wallis test, as appropriate in each case. For m x n contingency tables in which observed frequencies occupy *m* rows and *n* columns, a Fisher’s exact test was estimated using the Monte Carlo method. A two-tailed *p* < 0.05 was considered statistically significant. Statistical data clean-up and analysis were performed with the SPSS 22.0 program (IBM Inc. Chicago IL, USA).

## Results

**Fig. 1:**
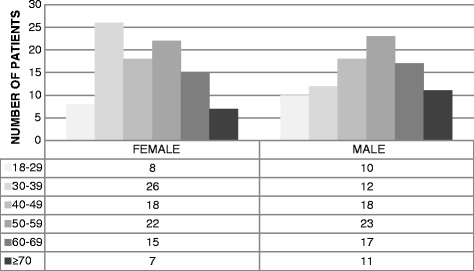
Distribution of HHT patients sorted by age and sex

Between 1 January 2005 and 31 December 2013, a total of 187 adult patients with HHT (96 women and 91 men) completed the descriptive scale and the visual analog scale (EQ-VAS) of the EuroQol 5D-3L tool. Distribution regarding age and sex is shown in Fig. 1. The average age of the patients was 48.8 years (SD: 14.8). Among the women, the average was 47.7 years (SD: 14.2). Among the men, the average age was 49.9 (SD: 15.4) (*p* = 0.29). Sixty six patients (35.3% of the total) were cases of HHT1; 57.8%, or 108 patients, were cases of HHT2. On 12 occasions, no mutations were identified (6.4%). A single patient presented a Smad 4 mutation (0.5%).

### Descriptive analysis considering health status dimensions

The health status results sorted according to the proportion of patients in each level of the five dimensions analyzed and by age subgroups showed that dimensions of pain/discomfort and anxiety/depression stood out the most among patients with HHT (47.6 and 43.9% of patients with problems, respectively). Meanwhile, self-care was the least affected dimension (14 patients, 7.5%). Patients over 60 years had the worst overall scores. The middle aged (aged 40 to 59) presented the biggest changes in their daily activities and in the level of their pain/discomfort. A worsening in the values considering all dimensions was observed when ageing of patients; this was significant in mobility (*p* < 0.0001), and nearly significant regarding self-care and anxiety/depresión (*p* = 0.06).

In terms of the presence of problems (levels 2 and 3) vs. absence of problems (level 1), in each dimension, we found that there was greater proportion of differences with age in the dimensions of anxiety/depression and pain/discomfort (Table 2).

**Table 2:** Percentage of patients with problems (yes) vs. absence of problems (no) in each of the EQ-5D dimension according to general characteristics

	Mobility		Self-care		Daily Activities		Pain/ Discomfort		Anxiety/Depression	
	No	Yes	No	Yes	No	Yes	No	Yes	No	Yes
Total (n) %	141 75.4	46 24.6	173 92.5	14 7.5	141 75.4	46 24.6	98 52.4	89 47.6	105 56.1	82 43.9
Age (yr) (%)										
18–29	100	0	100	0	84.6	15.4	76.1	23.9	73.1	26.9
30–39	92.3	7.7	75.1	24.9	47.8	52.2	58.2	41.8	62.9	37.1
40–49	57.5	42.6	57.9	42.1	62.1	37.9	47.7	52.3	52.2	47.8
50–59	47.3	52.7	53.2	46.8	44.5	55.5	48.8	51.2	45.0	55.0
60–69	24.5	75.5	22.5	77.5	35.2	64.8	38.4	61.6	34.8	65.2
>70	20.7	79.3	58.0	42.0	53.2	46.8	36.4	63.6	38.4	61.6
*P*-value*	<0.0001		0.06		0.14		0.11		0.06	

*Two-tailed Chi-squared test or Fisher exact test as corresponding

Elderly patients aged between 60–69 years exhibited the greatest deterioration in almost all dimensions. The exception was in daily activities, where the 50–59 and 60–69 age groups had the lowest score. In the dimension of self-care, the most affected population was also the 60–69 age group.

**Fig. 2:**
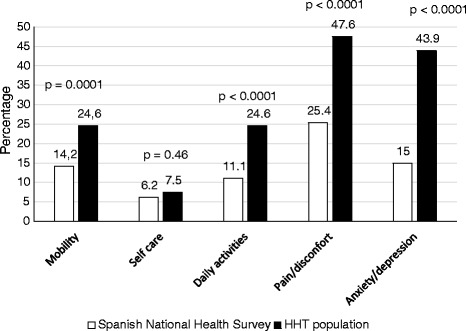
Comparison of percentages that report problems in each of the dimensions between HHT and Spanish health survey

Comparing the analysis of the HHT population with the 2011/2012 Spanish national health survey, HHT patients reported significantly more severe problems in all the descriptive system dimensions except in the self care dimension (Fig. 2).

In adition, the descriptive health status parameters of studies performed in Spain of the both general population and patients with chronic pathologies were compared (Table 3). Here, the EQ-5D-3 L was used in both the Catalonian health survey of 1995 and the Navarre general population health survey of 1995. In this case the differences between HHT patients and general population were statistically significant in all dimensions of EQ-5D.

**Table 3:** Comparison of the population of HHT with Spanish studies of the general population and patients with chronic diseases (%)

Eq-5d	Level^a^	General Population		Patients (general)	HHT Patients % (*N* = 187)
		Cataluña^10^ % (*N* = 12245)	Navarra^9^ % (*N* = 3000)	Chronical^10^ % (*N* = 120)	
Mobility	1	89.1	85.3	63.3	75.4
	2	10.7	14.7	36.7	24.6
	3	0.2	0	0	0
Self care	1	98.0	96.6	91.7	92.0
	2	1.8	3,4	8.3	8.0
	3	0.2	0	0	0
Daily Activities	1	93.6	89.0	85.8	75.4
	2	5.8	9.7	12.5	20.3
	3	0.6	1.3	1.7	4.3
Pain/Discomfort	1	74.2	70.6	42.5	52.4
	2	21.1	23.7	49.2	44.4
	3	4.7	5.7	8.3	3.2
Anxiety/Depression	1	86.3	82.2	62.5	56.1
	2	11.4	14.1	30.8	39.6
	3	2.3	3.7	6.7	4.3

^a^1 no problems, 2 some problems, and 3 significant problems

Comparing populations of individuals with chronic diseases with our sample of HHT patients, percentages showed similar results but mobility was more affected in the case of chronic conditions compared with HHT (*p* = 0.023) with a higher percentage of cases in level 2, while in the rest of dimensions the differences were not significant.

### Analysis of the visual analogue scale

**Fig. 3:**
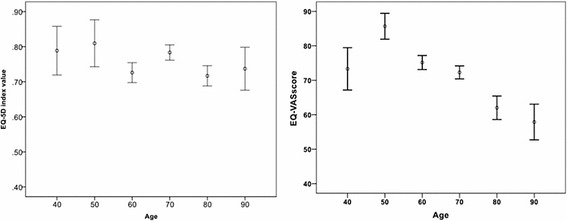
Distribution of VAS score and social index regarding age

The values of the visual analogue scale ranged from 0 to 100, with 0 being the worst subjective perception of health status, and 100 being the best. The mean VAS score was 73.7 (SD 19.0), and the median value was 80 (IQR 30). The scores were similar in men and women (median VAS score of 80 in both groups; *p* = 0.57). However, the VAS score worsened significantly with age generally (median score range from 90 in youngest aged group to 70 in oldest group; *p* = 0.001) and in the male group, in particular (*p* = 0.03), but not in female (*p* = 0.16) (Fig. 3). The scores were lower in patients with HHT 2 and in patients without an identified mutation (NO ID), although this difference was not statistically significant (Table 4).

**Table 4:** VAS values sorted by demographic and genetic characteristics

Variable	EQ VAS			
	*n*	Mean (SD)	Median (p25-p75)	*P*-value*
Sex				
Male	91	74.1 (19.8)	80 (65–85)	
Female	96	73.2 (18.1)	80 (60–90)	0.57
Age (yr)				
18–29	18	87.5 (10.9)	90 (80–96.25)	
30–39	38	79.7 (17.2)	85 (68.75-95)	
40–49	36	70.8 (19.3)	77.5 (55–83.75)	
50–59	45	69.3 (19.7)	75 (55–82.5)	
60–69	32	68.1 (18.4)	72.5 (52.5-80)	0.001
> 70	18	73.6 (19.3)	70 (58.75-92.5)	
HHT Type				
HHT1	66	75.7 (17.3)	80 (65–90)	
HHT2	108	73.7 (19.6)	80 (61.25-90)	
Smad4	1	20	20	
No ID	12	67.1 (15.3)	65 (51.25-80)	0.13

*Two-tailed independent samples Mann–Whitney *U* test or Kruskal-Wallis test as corresponding

In comparison to historical data, patients with HHT had higher mean VAS scores in perceived health status (73.7) compared with those obtained in the ESCA (71.3) and Navarre general population (71.6). Also reports about patients with chronic diseases showed lower means scores (63.9). However when comparing by age group with the EQ-VAS values from the 2011/2012 Spanish national health survey, patients with HHT presented lower values in all cases but in the oldest (>70) (probably biased because of the lower number of patients). This effect was significative in the groups aged between 35 and 54. The average values in the case of HHT were also lower than those of the general population (73.7 vs. 77.5).

### Social index value analysis

Social index values laid on in Table 5, showed a mean value of 0.77 (SD 0.19), with a median value of 0.79 (IQR 0.11). Distribution of results regarding age are shown in Fig. 3 with values ranging from 0.92 (youngest) to 0.65 (elderly). The differences in values were statistically significant in the case of the overall population (*p* < 0.001) and when stratified by sex (range from 0.92 to 0.61 from younger to older male patients, *p* < 0.001, range from 0.61 to 0.63 in women, *p* = 0.017). On the other hand, no significant differences were observed with respect to sex (median score of 0.76 in male and 0.79 in females; *p* = 0.86). With respect to genetics, patients with HHT type 1 or HHT type 2 had no significant higher scores than those without a mutation identified *(p* = 0.18).

**Table 5:** Social index values sorted by demographic and genetic characteristics

Variable	Social Index			
	*n*	Mean (SD)	Median (p25-p75)	*P*-value*
Sex				
Male	91	0.77 (0.20)	0.76 (0.69-1.00)	
Female	96	0.76 (0.19)	0.79 (0.69-0.80)	0.86
Age (yr)				
18–29	18	0.92 (0.12)	1.00 (0.80-1.00)	
30–39	38	0.83 (0.16)	0.79 (0.74-1.00)	
40–49	36	0.79 (0.20)	0.79 (0.71-1.00)	
50–59	45	0.74 (0.19)	0.74 (0.67-0.80)	
60–69	32	0.65 (0.20)	0.70 (0.59-0.79)	<0.001
> 70	18	0.70 (0.17)	0.72 (0.66-0.80)	
HHT Type				
HHT1	66	0.78 (0.17)	0.79 (0.70-1.00)	
HHT2	108	0.76 (0.21)	0.79 (0.68-1.00)	
Smad4	1	0.11	0.11	
No ID	12	0.73 (0.12)	0.74 (0.69-0.79)	0.18

*Two-tailed independent samples Mann–Whitney *U* test or Kruskal-Wallis test as corresponding

The comparison between the values for general populations and for people with chronic illnesses with the values for patients with HHT is presented in Table 6. The HHT had a lower score in comparison with the general population and one study of general patients (median score 1.00 vs. 0.79). However, this result is similar in the case series of patients with chronic pathologies (median score 0.78).

**Table 6:** Social index values of the HHT population in comparison to the studies in general population and the population of patients with chronic diseases in Spain

	Social Index				
	*n*	Mean	Median	Minimum	Maximum
General population					
Catalonia^10^	12245	0.89	1.00	−0.08	1.00
Navarre^9^	300	0.87	1.00	0.12	1.00
Cronical patients^10^	120	0.75	0.78	0.17	1.00
HHT patients	187	0.77	0.79	0.11	1.00

### Analysis of the subjective severity of the impact of epistaxis on quality of life

The impact of epistaxis on quality of life was classified either as mild, moderate or severe (Table 7). This did not vary according to sex (*p* = 0.66).

**Table 7:** Subjective perception of severity of epistaxis impact sorted by demographic and genetic characteristics

		Epistaxis subjective impact			
	*n*	Mild (%)	Moderate (%)	Severe (%)	*P*-value*
Sex					
Male	91	52.7	25.3	22.0	
Female	96	52.1	30.2	17.7	0.66
Age (yr)					
18–29	18	100.0	0.0	0.0	
30–39	38	60.5	31.6	7.9	
40–49	36	61.1	22.2	16.7	
50–59	45	37.8	35.6	26.7	
60–69	32	37.5	34.4	28.1	
> 70	18	33.3	27.8	38.9	0.001
HHT Type					
HHT-1	66	54.5	28.8	16.7	
HHT-2	108	49.1	28.7	22.2	
Smad4	1	0.0	0.0	100.0	
No ID	12	75.0	16.7	8.3	0.26

*Two-tailed Chi-squared test or Fisher exact test as corresponding

All patients of both sexes in younger ages (18–29 years) referred to the impact as mild. Meanwhile, the percentage of patients who reported a moderate and severe impact increased significantly with age in both (*p* < 0.001). There was no statistically significant difference in the impact of epistaxis with respect to genetics (*p* = 0.26).

On average, patients reporting a moderate to severe impact were older, with a mean age of 57.1 (SD 11.7) years in patients with severe impact and 52.5 (SD 12.4) years in them with moderate impact; mean age of patients with mild impact of epistaxis was 43.6 (SD 15.1) years, being this differences statistically significant (*p* < 0.0001). This effect held for women and men separately: (male: mild, moderate, severe respectively: 43.7 (16.1), 56.9 (11.7), 56.8 (11.0), *p* < 0.001 and female: 43.5 (14.1), 49.0 (12.0), 57.6 (14.2), *p* = 0.001).

### The relationship between parameters of quality of life and the subjective severity of the impact of epistaxis

In the case of both quality of life scales, EQ-VAS and the social index value of the EuroQol 5D-3 L, a statistically significant association was observed between the perceived severity of nasal epistaxis (nosebleeds) in HHT patients, and the repercussion of the epistaxis on their quality of life. The mean VAS score was 80.1, 74.5 and 55.5 for patients referred mild, moderate and severe impact of epistaxis respectively (*p* < 0.0001). Similarly, the mean social index values was 0.82, 0.78, 0.61 respectively (*p* < 0.0001). This effect held overall, and for women and men separately (men: 0.84, 0.76, 0.62; *p* < 0.0001); women: 0.81, 0.80, 0.59 (*p* < 0.0001).

## Discussion

The quality of life of patients is affected by HHT. This is due largely to the nasal epistaxis they suffer, which is generally the most prevalent symptom among this population. Other clinical features are bleeding from the gastrointestinal system and symptoms due to development of pulmonary, hepatic and brain arteriovenous malformations. Some studies on pharmacological or surgical interventions in patients with HHT apply quality of life scales (SF-36) as a variable in evaluating responses. Some examples are the studies of the effects of bevacizumab, N-acetylcisteine, and silicone nasal swabs on epistaxis [13–15]. However, few studies provide reference population data, health status standards, and general quality of life parameters. Furthermore, in the majority of publications, either the original or abbreviated SF-36 scale is applied. Examples are a Norwegian case series with 66 patients [16], an Italian case series with 50 patients [17], and a German study of 77 cases [18]. All these three studies had lower SF-36 scores excepting values in the category of pain, in two of the three studies. Also, in the third study, the worst score was reported in patients with gastrointestinal and hepatic symptoms. A German study performed on a population with HHT using the questionnaire of “Profile of quality of life of chronically ill patients” with an specific area for symptoms related with the condition, showed the epistaxis as the most disturbing symptom while HHT patients presented worse results in five of the six scales listed compared with patients with chronic pathologies as cardiomyopathy or rheumatic disease [9].

Despite attempts to create specific severity scales for patients with HHT, there are still no tools that are widely accepted. Only the HHT-Epistaxis Severity Score (HHT-ESS) [19] seems to be a promising indicator. This is due to its strong correlation with the SF-36 method. Even so, it is only valid as a measure of post-intervention monitoring of efficacy [20]. In 2011, a specific 13 item scale for estimating quality of life in HHT patients was proposed as complement to SF-36. However, this system is not yet validated, and is not widely employed [21]. Since 2005, the EQ-5D-3L questionnaire has been used for adult patients being treated in the HHT Unit [22]. EQ-5D-3L is a generic and standardized instrument created in 1990. It has been adopted because of its simplicity and its potential applications for post-intervention clinical efficacy studies as well as for estimations of quality-adjusted years of life. In 2009, the EuroQol Group launched a new version of the tool (EQ-5D-5L). However, in the present study, we used the original version and this was in order to be able to include the data from patients treated between 2005 and 2009. In the descriptive part of the present study and considering the five dimensions of the EuroQol, it was common for older patients to have a higher percentage of problems across the board in case series from the general population. Nevertheless, it is significant that the total HHT population had a higher than average level of problems in all dimensions, in comparison to data from the Spanish national health survey from 2011–2012 [23]. This was true all cathegories but self care. In HHT patients, anxiety/depression appears proportionally higher than in the general population, even though it was exceeded by the perception of pain/discomfort. High rates of clinical anxiety and depression in HHT patients have been reported, especially in women [24]. Therefore, there is a need for an appropriate system for prevention and treatment to be developed in this area.

Since the EuroQol 5D tool was introduced in Spain, various studies of the general population have been carried out with this tool. For example, EuroQol 5D was used in the health surveys of Catalonia and Navarre in 1995. It has also been used to study subgroups of patients with chronic and critical diseases. If we compare the percentage of problems reported by the HHT population analyzed with the available data from these populations, the data, as expected, fits neatly with that of the case series of patients with chronic pathologies. This result is consistent with the chronic nature and the penetrance of HHT symptoms. In the case of EQ-VAS, there are no significant differences according to sex or genetics but the oldest group showed a significant lower score. Moreover there are differences with the general population: the mean score is 3,86% lower in HHT data than in ENSE data. However, this score is higher than the one reported for the general population and for patients with chronic pathologies in previous studies. These data indicate that there may have been a bias in the data as a result of how patients are selected for treatment in the HHT Unit. For example, they may have been selected according to their ability to travel. On the other hand, the social index values in the HHT population were comparable with values from chronic patients. Thus, the social index value seems to be a more reliable tool for carrying out comparative studies. It is not possible to compare with the data from ENSE 2013. This is because the social index value is calculated using formula from the EuroQol 5D-5L. According to the social index value, age is associated with poor scores. This is particularly true in men, which is unusual, because, according to the ENSE, men tend to rate their health higher than women.

In order to acquire an additional indicator of the perception of quality of life perception that was specifically related to epistaxis, patients were asked to classify the impact of nosebleeds as mild, moderate, or severe. Once again, age was a primary factor. However the result was slightly more prominent in men. During their fertile years, women could have a hormonal ‘protective’ mechanism against epistaxis due to estrogen effects on nasal mucose wall thickening [25], coagulation improvement [26] and expression of endoglin in endothelial cells [27] while in men, the deleterious effects seem to be continuous and normally increase over time.

Finally, we examined the reliability of EuroQol 5D in estimating the impact on the quality of life of HHT patients of their most debilitating symptom. A significant correlation was found between the perceived severity of epistaxis (mild/moderate/severe), the EQ-VAS, and the social index value. This held true for the overall population and for the sexes separately. Therefore, they are valid methods for estimating the health status of this population regarding its main symptom. The genetics only influenced the quality of life results for HHT2 patients in the case of the lowest social index values. In support of a probable genetic influence, a prior study of a small number of patients postulated that one could distinguish between HHT2 and HHT1 patients. In the case of HHT2 patients, there was a bigger effect on the physical dimensions of SF-36, while with HHT1 patients, there was a greater effect in the area of mental health [28]. Recently, the quality of life of 127 Argentinian patients was evaluated using EuroQol 5D-3L. The average EQ-VAS score was 69 (ICR: 20.3). This was less than the EQ-VAS value of the Spanish population. This is likely because in this study, a higher percentage of patients reported having epitaxis (33.3% vs. 19.2%) [29].

## Conclusions

Pain/discomfort and anxiety/depression were the quality of life dimensions most altered in HHT patients with in Spain. There was an increase, with age, in the frequency of problems. Compared to the general population, patients with HHT had higher percentages of problems in the five dimensions analyzed. This result was similar in case series of patients with chronic diseases.

EQ-VAS quantitative values assigned by HHT patients were generally lower than those assigned by members of the general population. There were no significant differences between sexes. The score decreased according to age in the group as a whole. This result was most significant with men.

HHT patients had lower social index values than the general population. These values were comparable in case series of patients with chronic diseases. The score worsened significantly with age (in the group as a whole and in men).

Both male and female HHT patients who were older perceived the impact of nasal epistaxis on their quality of life as more severe.

There was a statistically significant association between EQ-VAS values and social index values of the perception of the severity of epistaxis. The EuroQol 5D-3L seems to be a valid tool for estimating the impact of epistaxis on the quality of life.

### Assessment of bias

Probable selection bias:

- Although the population studied included patients from practically the whole of the national territory, the majority originated from Cantabria and bordering communities.
- Patients from regions outside Cantabria who are admitted to the HHT Unit have a health status that allows them to travel. Therefore, there may have been a certain bias towards more moderate effects.
- Demographic characteristics of the groups of general populations and patients with chronic diseases analysed and compared with HHT population may vary specially in those studies with lower number of patients.

Probable information bias:

- The EuroQol 5D-3 L scale used to evaluate the health status in patients, has a ‘ceiling effect’ in its descriptive section used to distinguish between health statuses in relatively healthy populations. Also it cannot be applied in pediatric age groups.

Despite these possible limitations, the sample size and the overall characteristics of the sample appear to have been sufficiently representative. There was no noticeable observer bias, since it was always the same person who carried out the survey and documented the results.

## Abbreviations

*ACVRL1*: Activin A receptor type II-like 1
AVM: Arteriovenous malformations
ENSE: Spanish national health survey
ESCA: Catalonian health survey
ESS: Epistaxis severity score
Euroqol 5D-3L: Euroqol five dimensions-three levels
HHT: Hereditary haemorrhagic telangiectasia
ICD: International classification of disease
INE: Spanish National Institute for statistics
SF-36: Short form-36 Health Survey
VAS: Visual analog scale
